# Timing insights in losartan-induced pancreatitis: a clinical case report

**DOI:** 10.1093/omcr/omag025

**Published:** 2026-03-23

**Authors:** Suraj Karanjit, Nistha Shahi, Kul Raj Shahi, Dipendra Chapagain, Jay Bhushan Jha, Jeevan Gyawali

**Affiliations:** Department of Internal Medicine, Rural Health Education and Service Center, Kathmandu 44600, Nepal; Department of Internal Medicine, Karnali Province Hospital, Birendranagar, Surkhet 21700, Nepal; Department of Internal Medicine, Karnali Province Hospital, Birendranagar, Surkhet 21700, Nepal; Department of Internal Medicine, Patan Academy of Health Sciences, School of Medicine 44700, Nepal; Research Unit, Nepal Medical Association 44600, Nepal; Department of Internal Medicine, Chirayu National Hospital and Medical Institute (CNHMI), Kathmandu 44600, Nepal

**Keywords:** acute pancreatitis, drug-induced pancreatitis, hypertension, losartan, supportive

## Abstract

Acute pancreatitis (AP) is a gastrointestinal disorder frequently associated with gallstones, hypertriglyceridemia, and alcohol use. Drug-induced pancreatitis has been documented in the medical literature, with various medications implicated as potential triggers. We report a case of a 31-year-old man with hypertension who presented with severe upper abdominal pain and was diagnosed with AP. Extensive diagnostic evaluation excluded common causes, pointing towards losartan. Symptomatic management and cessation of losartan led to improvement. This is a rare instance of losartan-induced AP. The patient experienced symptoms after a year of use, suggesting that pancreatitis can occur at any time during therapy. The underlying mechanisms are unclear; however, they might include decreased bradykinin breakdown leading to localized pancreatic duct angioedema. This highlights the importance of differentiating drug-induced pancreatitis from idiopathic causes. This case highlights the importance of considering pancreatitis in differential diagnosis, particularly in patients lacking typical risk factors.

## Introduction

Acute pancreatitis is a common gastrointestinal disorder encountered in clinical practice. It is typically caused by factors such as gallstones, hypertriglyceridemia, excessive alcohol consumption, and iatrogenic causes like endoscopic retrograde cholangiopancreatography (ERCP). Other contributing factors include hypercalcemia and certain medications, including antiretrovirals, immunosuppressants, diuretics, and antibiotics [1].

Between 1968 and 1993, the WHO received reports of 525 different drugs from various substance classes suspected of inducing pancreatitis as an adverse effect [2]. Recent studies show only 141 drugs were identified as causing acute pancreatitis, but only 106 drugs have been published in the literature as causing acute pancreatitis [3]. Although drug-induced pancreatitis is relatively rare, it is generally mild and self-limiting in most cases [2]. Angiotensin-converting enzyme (ACE) inhibitors are infrequently implicated in causing pancreatic inflammation. Angiotensin II receptor blockers (ARBs), such as losartan, which are used for similar indications as ACE inhibitors, tend to have a more favorable side-effect profile [4].

Our clinical case demonstrates that losartan is the cause of pancreatitis, following the exclusion of all other potential causes through comprehensive laboratory tests and imaging studies. Many cases in the literature report that pancreatitis occurs within a few days or weeks of starting losartan; however, our case demonstrates that pancreatitis can occur at any time during losartan therapy, even after a year of treatment. This case report has been reported as per the Surgical CAse REport (SCARE) guidelines [5].

## Case presentation

A 31-year-old man with a history of hypertension arrived at the emergency department of primary care with severe upper abdominal pain lasting one day. The pain was severe, centered in the epigastric region, radiating to the back, and accompanied by nausea. There were no relieving factors, and there was no history of smoking, alcohol use, or recreational drug use. His medications included Amlodipine 5 mg and Losartan 25 mg, both taken regularly for the past year.

Initially symptomatic treatment was done, and the patient was subsequently admitted to the High Dependency Unit (HDU) at a tertiary care center. On admission, he appeared unwell but remained oriented and afebrile, with vital signs mostly within normal limits except for an elevated blood pressure of 150/100 mmHg. Physical examination revealed tenderness in the right upper quadrant, with no other significant findings.

Laboratory investigations showed a white blood cell count of 13 400/cu mm with 90% neutrophils, indicating leukocytosis. Serum C-reactive protein (CRP) was tested and found to be elevated at 132 mg/l. His serum alpha-amylase level was elevated at 1260 IU/L (normal range: 25-240 IU/L), while his lipase level was elevated at 315 IU/L (normal range: 0-60 IU/L). Arterial blood gas (ABG) analysis was normal with a serum lactate level of 0.7 mmol/l and a pH of 7.508. Other blood tests, including hematological parameters, electrolytes, cholesterol, triglycerides, liver function tests (ALT 45 IU/L and AST 30 IU/L), and calcium levels, were within normal limits.

Abdominal ultrasonography (USG) revealed a normal-sized pancreas with decreased echogenicity. The liver appeared slightly enlarged (~15.7 cm) with mildly increased echogenicity but maintained a normal shape and outline. Ultrasound of the gallbladder showed no gallstones, and the pancreatic duct appeared normal. Additionally, abdominal ultrasound identified mild ascites.

A chest X-ray demonstrated bilateral pneumonia with a right-sided pleural effusion.

**Figure 1:**
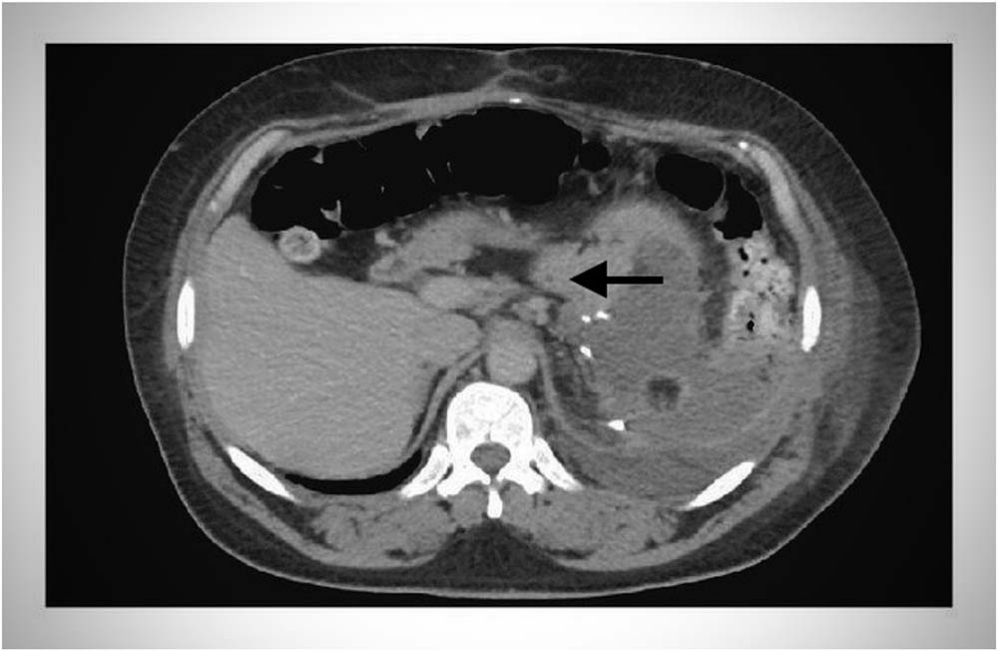
CT scan of abdomen showing fluid collection around the tail of the pancreas (black headed arrow).

CT-scan-abdomen showed normal enhancing pancreas with swelling and fluid collection around the tail of the pancreas (Fig. 1).

The patient had a normal lipid profile, normal hepatitis B and C serologies, normal calcium and cholesterol levels, no history of trauma and recent ERCP, no family history of pancreatic disorder, and autoimmune markers and viral serologies (e.g. mumps, CMV) were not tested due to unavailability. Based on the temporal association of symptoms and exclusion of other etiologies, losartan was suspected as the likely cause of his pancreatitis, despite its rarity. No formal consultation was conducted with the pharmaceutical company in this case.

The patient was managed symptomatically, and losartan was discontinued. He continued on Amlodipine 5 mg for hypertension management. Over the hospitalization period, his condition improved significantly, with amylase levels normalizing to 131 IU/L and lipase levels to 210 IU/L by the third day post-discontinuation of losartan. He was discharged with a plan for follow-up at the primary care center in two weeks. While a re-challenge with losartan to confirm causality was not performed due to ethical concerns, the clinical improvement post-discontinuation supports a probable causative role of losartan.

The patient was followed up at a two-week interval. During this period, the patient showed significant improvement in symptoms. Repeat tests of serum alpha-amylase and lipase levels showed values of 50 IU/L and 30 IU/L, respectively, indicating a return to normal levels.

Given the improvement and recognizing losartan as the suspected agent, it was discontinued. The patient was pleased with the treatment results and reported a significant improvement in their overall condition.

## Discussion

The World Health Organization(WHO) database identifies 550 medications as potentially contributing to Drug-Induced Acute Pancreatitis(DIAP) [2]. DIAP is a rare etiology of acute pancreatitis occurring in 2% of the general population [6]. The actual incidence of this condition is currently unknown. However, causation has been documented based on the documented cases [1].

Previous literature has documented a few cases of ARB-induced pancreatitis as Anwar et al. reported a similar case where losartan was implicated as the cause of pancreatitis after excluding other potential causes [1]. Similarly, Birck et al. described a patient who developed pancreatitis after starting losartan, with resolution upon discontinuation of the drug [4]. However, many of these cases report that pancreatitis occurs within a few days or weeks of starting losartan. In contrast, our patient had been taking losartan for a year before developing pancreatitis. This indicates that pancreatitis can occur at any time during losartan therapy, not just shortly after initiation, which is an important consideration for physicians. The underlying mechanisms remain speculative. This table highlights the variability in latency periods and the importance of drug discontinuation in recovery (Table 1).

**Table 1:** Comparison of literature.

Author	Age/Sex	Drug	Onset	Enzymes	Imaging	Rechallenge	Outcome
Anwar et al. [1]	45/M	Losartan	10 days	↑Amylase, ↑Lipase	Pancreatic edema	No	Resolved
Birck et al. [7]	58/F	Losartan	2 weeks	↑Amylase	Normal	No	Resolved
Current case	31/M	Losartan	1 year	↑↑Amylase, ↑Lipase	Mild changes on USG	No	Resolved

Angiotensin type 1 (AT-1) receptor binding of the chemical angiotensin II is inhibited by angiotensin receptor blockers (ARBs). These receptors are G-protein coupled receptors; AT-2, AT-4, and MAS are the other three categories [8]. A possible mechanism for angiotensin-converting enzyme (ACE) inhibitor–induced acute pancreatitis involves local angioedema of the pancreatic duct [9]. ACE inhibitors reduce the degradation of bradykinin, which is associated with the development of angioedema [10]. Studies indicate that bradykinins are released during acute pancreatitis, consistent with the observed increased vascular permeability in the pancreas during this condition [10]. This release can lead to pancreatic edema, trapping enzymes and other toxic substances within the pancreas and causing tissue damage and acute pancreatitis [10]. Additionally, angiotensin II receptors may play a crucial role in regulating secretion and microcirculation within the pancreas [11].

Medications associated with drug-induced acute pancreatitis (DIAP) are categorized into four classes based on reported cases [12]. Class I includes medications with at least one reported case of DIAP. Class IA consists of drugs suspected to cause DIAP after excluding the most common causes of acute pancreatitis. Class IB includes medications identified as the cause of DIAP following a re-challenge when other common causes could not be ruled out. Class II includes medications with a latency period of 75%. Class III drugs show no latency, and Class IV comprises medications with very few reported cases that do not fit into other classes [12]. Among renin-angiotensin system (RAS) inhibitors, drugs such as captopril, ramipril, enalapril, lisinopril, quinapril, benazepril, losartan, and telmisartan are mainly associated with DIAP and are classified as Class IB.

DIAP is typically diagnosed only after excluding common causes such as gallstones, alcoholism, post-trauma, and post-endoscopic retrograde cholangiopancreatography (ERCP). Once these common causes are ruled out, the diagnosis is based on clinical symptoms, elevated serum lipase and amylase levels, abdominal imaging, and the recurrence of symptoms upon reintroduction of the suspected medication [11].

The role of Amlodipine must also be considered. Though rare, calcium channel blockers have been implicated in pancreatitis, due to alterations in pancreatic blood flow or direct toxic effects [11]. However, continued use of Amlodipine in our patient with no recurrence of symptoms supports losartan as the more likely offending agent.

Based on our extensive literature review, we have identified previous instances where losartan has been implicated in causing pancreatitis [4, 13, 14]. Notably, there have been documented cases where patients experienced recurrent pancreatitis upon re-challenging with losartan, underscoring its potential role as a causative agent in this serious medical condition. Pancreatitis is known to have significant morbidity and mortality rates, emphasizing the importance of further investigating the safety profile of commonly prescribed medications like losartan. Given the rarity of drug-induced acute pancreatitis (DIAP), healthcare providers should maintain a high level of clinical suspicion and awareness regarding these uncommon side effects associated with medications such as losartan.

A limitation of this case report is the challenge in definitively establishing causality between losartan and pancreatitis. While the temporal association and clinical improvement upon discontinuation strongly suggest losartan as the causative agent, other factors cannot be entirely ruled out. Further research, particularly larger controlled studies, is necessary to accurately determine the incidence and elucidate the mechanisms underlying ARB-induced pancreatitis. An enhanced understanding of the pathophysiology and development of more precise diagnostic criteria would significantly enhance the management and prevention strategies for such cases.

## Conclusion

In conclusion, this case highlights the importance for healthcare providers to include drug-induced pancreatitis in their differential diagnosis of acute pancreatitis, particularly in patients lacking common risk factors such as gallstones or alcohol use. Notably, this case demonstrates that pancreatitis can occur at any time during losartan therapy, even after prolonged use, highlighting that the onset of drug-induced pancreatitis is not limited to the initial period after starting the medication. Early recognition and prompt cessation of the suspected offending drug are essential for achieving favorable clinical outcomes.
